# The First Word Recalled Measure – A Potential Addition to Clinical Exams

**DOI:** 10.3389/fneur.2021.561824

**Published:** 2021-02-01

**Authors:** Irit Shapira-Lichter, Noga Oren, Anita Asvadurian, Rachel Ben-Hayun, Tali Fisher, Judith Aharon-Peretz, Amir Glik

**Affiliations:** ^1^Functional MRI Center, Beilinson Hospital, Petach Tikva, Israel; ^2^Sackler Faculty of Medicine, Tel Aviv University, Tel Aviv, Israel; ^3^Cognitive Neurology Clinic and Department of Neurology, Beilinson Hospital, Petach Tikva, Israel; ^4^Cognitive Neurology Institute, Rambam Health Care Campus, Haifa, Israel

**Keywords:** memory, neuropsychological tests, Alzheimer disease, mild cognitive impairment, differential diagnosis

## Abstract

Characterizing episodic memory abilities is highly important in the diagnosis of Alzheimer's disease (AD) and mild cognitive impairment (MCI), and usually includes wordlist learning and recall tasks. Clinical evaluations typically focus on the number of words recalled, ignoring additional information, like serial position. Here, we tested the potential value of two serial positioning measures for clinical diagnosis – how retrieval is initiated, as measured by the first word recalled, and how it proceeds – using data from patients with AD and MCI that completed a wordlist learning and recall task. Our results show that during the early stages of learning, patients with AD are less prone to retrieve the first word from the wordlist, manifested as lower primacy effect in the first word recalled, compared with MCI patients. The first word recalled measure adds to the differentiation between the groups over and above the total number of words learned. Thus, the first word recalled during word list learning and recall tasks may be used as a simple complementary measure to distinguish between MCI and AD during standard neuropsychological evaluations.

## Introduction

Wordlist learning and recall tasks are prevalent in neuropsychological evaluations. They examine short- and long- term memory, learning ability, retrieval initiation and retrieval dynamics. Performance in these tasks is hampered in conditions like healthy aging (1) Parkinson's disease (2), geriatric depression (3), Alzheimer's disease (AD) (4), and mild cognitive impairment (MCI) (5–7). Each condition has a unique pattern of deficits (8, 9), which can be used for differential diagnosis and prognosis at the single participant level. Typical clinical evaluations focus on the number of words recalled, ignoring the information embedded in the identity of the retrieved words and their serial position.

Retrieval of items from a list is related to their serial position. Primacy and recency are key serial position effects: higher recall rates of words positioned at the beginning and end of the list, compared with middle list words (10, 11). They rely on distinct and independent capabilities (12). Primacy effect results from the longer rehearsal time available for the first items on the list and reflects long term memory, while recency effect relays on retrieval of the very last items from short term memory (11). In the clinic, this dissociation between short- and long- term memory abilities is manifested as spared serial position effects in healthy aging, despite a typical reduction in total recall (1, 13); while patients with dementia or MCI (3, 14) and cognitively normal individuals at risk to develop dementia (15) demonstrate decreased primacy effect along with spared recency effect up until severe dementia (14).

Earlier studies used measures of serial position scores to discriminate MCI from healthy controls (16–18), amnestic from non-amnestic MCI (19), MCI from patients with AD (20) and patients with frontotemporal dementia from patients with AD and vascular dementia (21). These scores also predict the conversion from MCI to AD (22). Nevertheless, these measures failed to outperform the standard clinical measures used for differential diagnosis (16). Yet there are other serial positioning measures proven informative in cognitive psychology literature (23) that were not applied in clinical populations: (1) the first word recalled; (2) recall temporal contiguity. We tested the hypothesis that these measures may refine differential diagnosis between AD and MCI, above and beyond the standard clinical measures.

The first word recalled measure describes the first word that was recalled by the participant in terms of its serial position on the list during encoding (23, 24). This measure is easily acquired and indicates how retrieval is initiated. In immediate free recall there is a prominent recency effect in the first word recalled, as recent words are more likely to be recalled as the first item in participants' output sequence (23, 25). In delayed free recall, the recency effect of the first word recalled is attenuated (23). This effect is also related to list length, with longer lists showing stronger recency effect and shorter list showing stronger primacy effect (26).

Recall temporal contiguity refers to the probability of two successively recalled items to come from neighboring list positions (27). This measure captures the dynamics of the retrieval process and the inter-item associations created based on temporal position. Using a temporal contiguity measure termed lag- contiguity Response Probabilty (lag-CRP), young participants show a tendency to retrieve items from nearby serial position of the just-recalled word as well as asymmetry, as forward transitions are about twice as likely as backward transitions (23). In immediate free recall, the effect depends on the output position, whereas in delayed recall, the effect is stable throughout the output sequence.

Younger and older participants have a similar pattern in the first recalled word measure, indicating that retrieval is initiated in a similar manner regardless of age (28). The groups differ in lag-CRP with more flatten lag-CRPs for older adults, indicating that with age, recall transitions reflect the inter-item temporal relations less. Based on these studies, we assume that patients with AD will show reduced primacy effect in the first word recalled and reduced temporal contiguity effects. As far as we know these two measures were not studied in patients with MCI or AD.

## Materials and Methods

A retrospective comparative cohort study design was used, waiving the need for informed consent. The data collection and analysis protocols were approved by the local Institutional Review Boards. All subjects were included anonymously.

### Participants

The study included data of patients examined in the Cognitive Neurology Clinics at Rabin Medical Center and at the Rambam Health Care Campus between 2010 and 2017. They completed the Consortium to Establish a Registry for Alzheimer's Disease (CERAD) test and the Mini–Mental State Examination (MMSE) as part of their evaluation and were diagnosed with AD or MCI. All subjects performed neurocognitive evaluation which included executive, memory, language and visuospatial domains (data not presented). Diagnosis was ascertained using the 2011 guidelines of the National Institute on Aging-Alzheimer's Association Workgroups (29, 30). MCI patients were of the amnestic multiple domain subtype with memory impairment as the main deficit. Patients with trisomy 21, traumatic brain injury, HIV infection, previous encephalitis or s/p brain surgery or other types of dementia (vascular, frontotemporal, Lewy body or Parkinson's disease) were excluded. The study included 199 patients with AD (72 males, 127 females, average age 78.7 y/o, range 55–96 y/o, average MMSE score 19.98, range 10–29) and 35 patients with MCI (15 males, 20 females, average age 74.9 y/o, range 61–90y/o, average MMSE score 26.7, range 23–30).

### The Wordlist Learning and Recall Task

A modified Hebrew version of a wordlist learning and recall task from the CERAD battery was used (31). It includes three consecutive encoding and free recall trials, followed by delayed recall and recognition trial. During encoding, a single list of ten unrelated concrete and emotionally neutral words was presented in a constant order. The list's length was suited to the patients' condition that may become distressed by longer lists (32). Patients overtly retrieved as many words as possible during free and delayed recall. Retention interval for delayed recall was 5 min filled by visuospatial tasks. Performance in the recognition trial is out of our scope.

### Measures and Statistical Analysis

***Immediate recall***: total number of correct recall in the first trial. ***Delayed recall***: total number of correct recall in the delayed recall trial. ***Corrected total learning***: total number of words recalled in the three learning trials minus three times the number of words recalled in the first trial. ***Learning rate***: subtracting the number of words recalled in first recall trial from the number of words recalled in the third recall trial. ***Forgetting rate***: number of words recalled on the delayed recall trial minus the number of words recalled on the third recall trial. *T*-tests were used to compare the groups in each measures.

***Serial position curves***: the probability of recall for each serial position in the encoded wordlist. The plots provided descriptive statistics while the serial position effect measures were tested qualitatively.

***First recalled word***: the first response in each free recall trial. Responses were classified as “First” (the very first word in the encoded wordlist, *word 1*), “Recent” (one of the two last words, *words 9 or 10*) or “Middle-list” (*words 2–8*). Lack of any recall was classified as “None.” Primacy was restricted to the first item in the wordlist since it is unique and qualitatively different from the following items (23, 33). We examined primacy or recency effects in the first word recalled, manifested as above chance proportion of “First” and “Recent” responses, respectively. To this end, Chi-square goodness-of-fit tests were used for each group and recall trial. These tests examined whether the observed distribution of first recalled word was significantly different from a uniform distribution of expected probabilities of 0.1, 0.2, and 0.7 for “First,” “Recent,” and “Middle-list” responses, respectively. Clusters were used to minimize the number of comparisons thus avoiding the statistical caveats associated with multiple comparisons. William's correction was applied for cells with an expected count of <5 patients. Bonferroni correction for multiple comparisons was used. Standardized residual scores were used as *post-hoc* measures, so scores larger than 1.96 for either “First” or “Recent” indicate a significant primacy/recency effect, respectively. Comparing the groups in each learning trial was done using Chi-square tests of independence. Here, there was no need for correction, since <20% of cells had an expected count of <5 patients. Bonferroni correction for multiple comparisons was used. Standardized residual scores were used as *post-hoc* measures that reflect the contribution of specific cells to the differences between groups. Binary logistic regression with nested models examined whether the first word recalled measure adds to the groups differentiation, over and above the standard measure of the number of words recalled.

***Recall temporal contiguity***: probability of recalling an item as a function of its lag from the item that was retrieved in the previous trial (27). A lag is the distance in the encoded wordlist between the current and previous retrieved word. To compute this measure, the number of actual lag was calculated and normalized by the number of transition this participant performed in this trial. The normalization system used by Quenon et al. (34) was employed since it yields a temporal contiguity measure that deals well with the minimal data available per condition/participant and is relatively insensitive to potential group-differences in overall recall level. The recall temporal contiguity measures were constructed only for the delayed recall trial, since these measure are known to change along the retrieval process in immediate recall and accordingly, different lag-CRP patterns are expected for each output position (i.e., for the 1st, 2nd, 3rd etc. word retrieved) (23). Consequently, creating a curve per participant requires multiple wordlists, which is not feasible in a regular clinical examination. In contrast, in delayed recall, lag-CRP is stable across the output positions, making it possible to average across the output positions and to calculate the measure from a single wordlist. To summarize, in the recall temporal continuity measures the focus was on the delayed recall data, and Quenon's et al. (34) normalization method was applied.

Following Quenon et al. (34) the groups were compared in three measures of recall temporal contiguity:

(1) Nearby forward transition (lag = 1);

(2) Nearby backward transition (lag = −1);

(3) A transitive associations index that compared short (lag = ± 2) vs. long-distance (lag >4 or < −4) transitions and was computed as follows:

$$\begin{array}{l} \frac{\left[F\left(-2\right)+F\left(+2\right)\right]-\left[\sum_{i=-10}^{-5}Fi+\sum_{j=+5}^{+10}Fj\right]}{\left[F\left(-2\right)+F\left(+2\right)\right]+\left[\sum_{i=-10}^{-5}Fi+\sum_{j=+5}^{+10}Fj\right]} \end{array}$$

[F(-2)+F(+2)] denotes the proportion of transitions at |lag|=2.

$\left[\overset{-5}{\underset{i=-10}{\sum }}Fi+\overset{+10}{\underset{j=+5}{\sum }}Fj\right]$ denotes the proportion of transitions to remote lags (i.e., |lag|>4).

The analysis included patients that retrieved at least two items during delayed recall – i.e., have at least a single lag (17 AD; 26 MCI). The hypothesis that patients with AD will have lower scores as compared with patients with MCI, reflecting impaired continuity processes, was tested using independent sample *t*-tests.

## Results

### Number of Words Recalled

Patients with MCI outperformed patients with AD in immediate recall, corrected total learning and delayed recall (Table 1). There was no significant difference between the groups in learning rate and forgetting rate.

**Table 1:** The number of words recalled per group and group comparisons in the standard measures used in clinical evaluations.

**Measure**	**Mean (std)**		***t*-test**
	**MCI**	**AD**	
Immediate recall	3.97 (1.27)	2.4 (1.35)	*t*_(232)_ = −6.44, *p* < 0.001
Corrected total learning	4.23 (2.43)	2.66 (3.15)	*t*_(231)_ = −2.81, *p* < 0.005
Delayed recall	3.17 (2.3)	0.47 (1.16)	*t*_(38)_ = −6.82, *p* < 0.001
Learning rate	2.37 (1.5)	1.66 (2.5)	*t*_(231)_ = −1.64, *p* = 0.103
Forgetting rate	−3.17 (1.87)	−3.56 (2.7)	*t*_(231)_ = 0.81, *p* = 0.417

### Serial Position Effects

**Figure 1:**
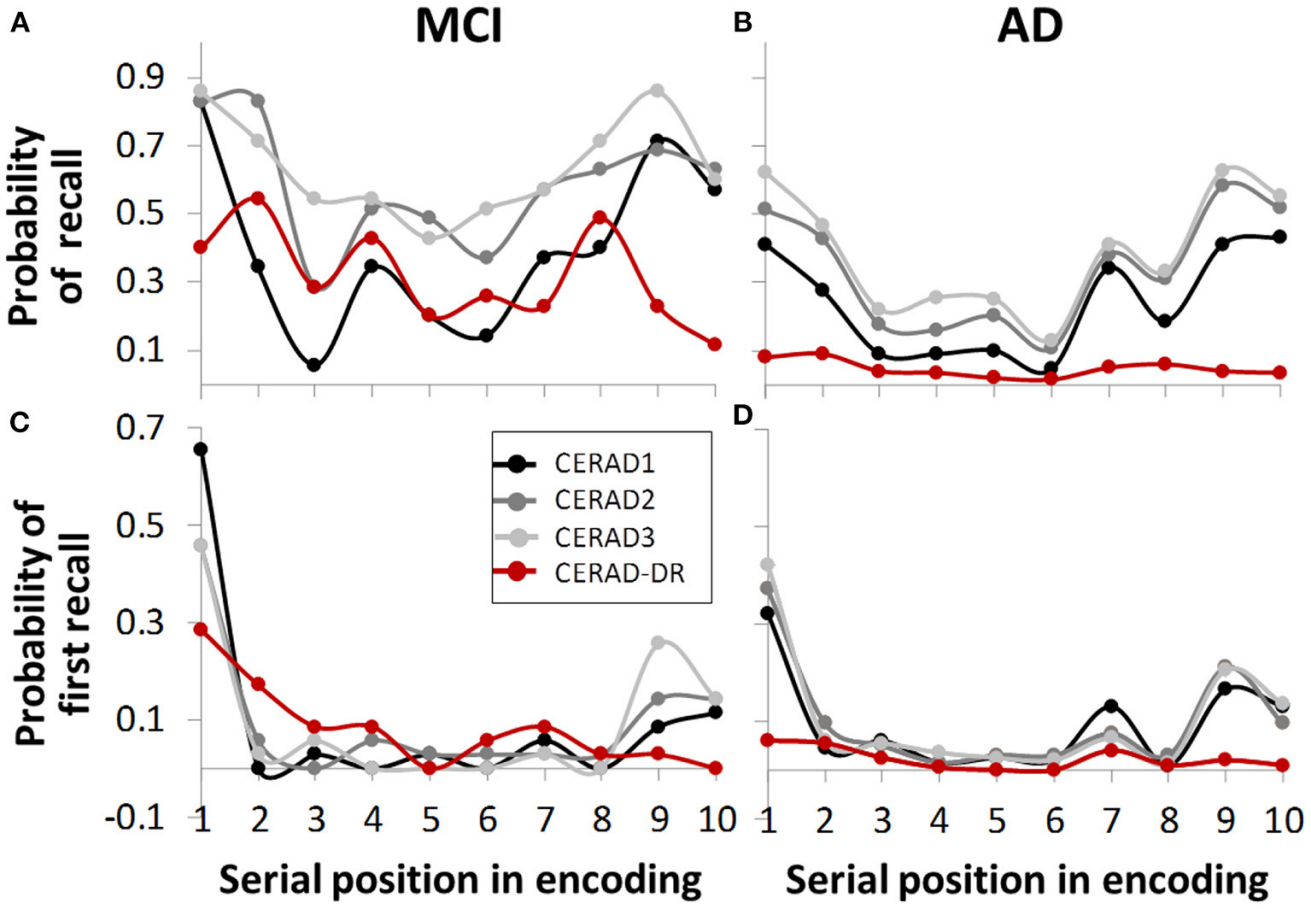
Serial position curve, representing retrieval chances as a function of serial position in the wordlist during encoding. Graphs **(A,B)** show overall probabilities, **(C,D)** show probabilities for first word recalled. In immediate recall (CERAD 1, 2, 3, gray-scale graph lines), both groups showed primacy and recency effects in both overall probability **(A,B)** and first word recalled **(C,D)**. In delayed recall (CERAD-DR, red), none of the groups showed recency effect **(C,D)**, as expected. In contrast, there is a seemingly considerable reduction in the dementia group in primacy effect during delayed recall, as compared to other learning trials **(D)**, which is less evident in the MCI group **(C)**.

Standard serial position curves showed that performance improved as learning progressed, manifested as higher and flatter curves as the list was re-learned (Figures 1A,B). The figures further suggests possible primacy and recency effects in both groups and three learning trials, manifested as greater probability to recall first and last words, respectively (CERAD 1-3). In delayed recall, there was no recency effect, as anticipated (Figures 1A,B).

### First Word Recalled

There were significant serial position effects for both groups in all recall trials (Table 2; Figures 1C,D). *Post-hoc* analyses show significant primacy effect for both groups in all trials. Patients with AD had significant recency effect in trials 1-3 but not in delayed recall. Patients with MCI had significant recency effect only in the third recall trial.

**Table 2:** Primacy and recency effects, calculated using the first word recalled measure.

		***N***				**Chi square**	**Posthoc analysis: standardized residual**		
		**F**	**M**	**R**	**None**		**F**	**M**	**R**
CERAD 1	AD	65	60	59	15	*c*^2^(2) = 168.162, ***p*** **<** **0.008** ^+^	**10.9**	**−6.10**	**3.70**
	MCI	23	4	8	0	*c*^2^(2) = 123.585, ***p*** **<** **0.008** ^+^^*^	**10.42**	**−4.14**	0.38
CERAD 2	AD	74	59	61	5	*c*^2^(2) = 209.803, ***p*** **<** **0.008** ^+^	**12.40**	**−6.60**	**3.60**
	MCI	17	8	10	0	*c*^2^(2) = 63.264, ***p*** **<** **0.008** ^+^^*^	**7.22**	**−3.33**	1.13
CERAD 3	AD	84	46	68	1	*c*^2^(2) = 290.398, ***p*** **<** **0.008** ^+^	**14.40**	**−7.90**	**4.50**
	MCI	16	4	15	0	*c*^2^(2) = 69.613, ***p*** **<** **0.008** ^+^^*^	**6.68**	**−4.14**	**3.02**
CERAD DR	AD	12	26	6	155	*c*^2^(2) = 14.546, ***p*** **<** **0.008** ^+^^*^	**3.60**	–0.90	–0.90
	MCI	10	18	1	6	*c*^2^(2) = 21.130, ***p*** **<** **0.008** ^+^^*^	**4.17**	–0.51	–1.99

*For each trial, primacy and recency effects were examined for AD (N = 199) and MCI (N = 35) patients, via a comparison to a uniform probability (0.1, 0.7, 0.2, respectively). Standardized residuals were used to identify the cells which underlie the significant results (i.e., statistically significant cells are those with absolute standardized residual > 1.96, indicated in bold fonts). F, first word (word 1); M, middle list words (words 2–8); R, recent words (words 9–10); N, number of words within each cell; DR, delayed recall*.

* *William's correction was utilized since a cell with expected count <5 was identified*.

+ *Bonferroni correction for multiple comparisons*.

Group comparison showed stronger primacy effect for the MCI group in the first recall trial and not in the second and third recall trials (Table 3). To test whether the group difference was due to disease severity and general cognitive ability, as reflected by the MMSE score, the analysis was repeated with a subgroup of patients with MMSE score of 25 or more; performance which is considered normal. The groups were matched for age [AD: n=31, averaged age 75.4 y/o; MCI: *n* = 31, averaged age 74.2 y/o; *t*_(60)_ = 0.75, *p* = 0.456]. Due to the small subgroups, the “Recent” and “None” categories were united and compared to the “First” category. A Chi-square test of independence replicated the result of stronger primacy effect for the MCI group in the first recall trial [$\chi_{\left(1\right)}^{2}$ = 4.239, *p* < 0.05].

**Table 3:** Primacy and recency effects, calculated using the first word recalled measure.

		**N**				**Chi square**	**Posthoc analysis: standardized residual**			
		**F**	**M**	**R**	**None**		**F**	**M**	**R**	**None**
CERAD 1	AD vs. MCI	65	60	59	15	*c*^2^(2) = 12.0875, ***p*** **<** **0.01** ^+^	−1.04	0.85	0.36	
		23	4	8	0		**2.38**	**−1.95**	−0.83	
CERAD 2	AD vs. MCI	74	59	61	5	*c*^2^(2) = 1.469, *p* > 0.05 ^+^				
		17	8	10	0					
CERAD 3	AD vs. MCI	84	46	68	1	*c*^2^(2) = 2.611, *p* > 0.05 ^+^				
		16	4	15	0					
CERAD DR	AD vs. MCI	12	26	6	155	*c*^2^(1) = 0.432, *p* > 0.05 ^+^				
		10	18	1	6					
		44			155	*c*^2^(1) = 51.172, ***p*** **<** **0.005** ^+^	**-2.30**			1.50
		29			6		**5.50**			**-3.70**

*Primacy and recency effects were directly compared between the groups. In the delayed recall only, the probability of no recalls was also compared. Standardized residuals were used to identify the cells which underlie the significant results (i.e., statistically significant cells are those with absolute standardized residual > 1.96, indicated in bold fonts). F, first word (word 1); M, middle list words (words 2–8); R, recent words (words 9–10); none, no word was retrieved; N, number of words within each cell; DR, delayed recall*.

+ *Bonferroni correction for multiple comparisons*.

**Figure 2:**
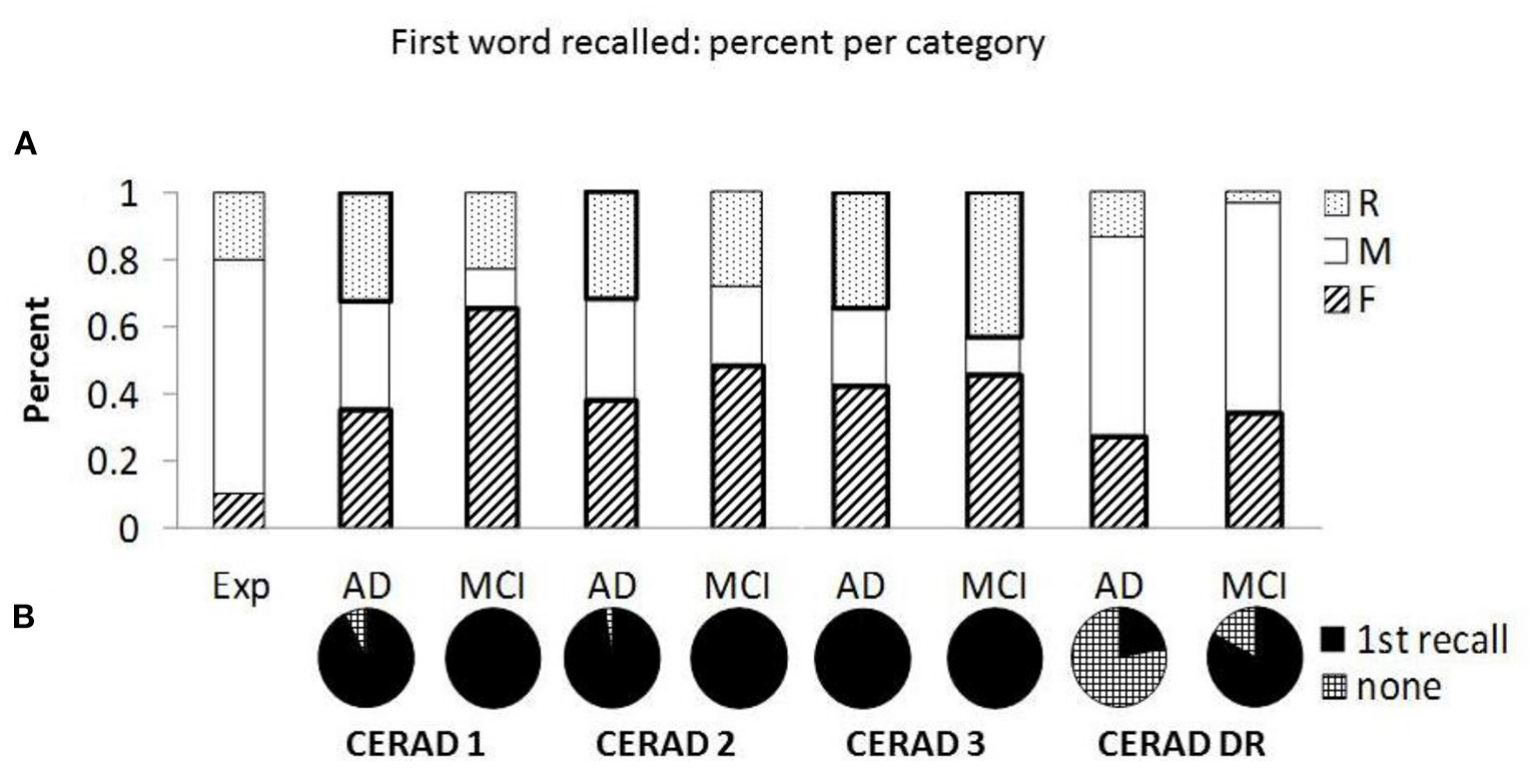
The distribution and proportion of first word recalled. **(A)** Presents the percent of “First” (F), “Middle-list” (M), and “Recent” (R) responses retrieved as the first word, relative to the expected percent based on uniform distribution (see the “Exp” bar on the left). The percent is calculated after discarding “None” responses. Sections denoted with thick borders indicate significant difference from the expected distribution. **(B)** Presents the proportion of “None” responses (checkered) relative to recalls (first recall, black) in each group and recall trial [CERAD 1-3 and delayed recall (CERAD-DR)].

Comparing the groups in the delayed recall trial was done after collapsing the “Middle-list” and “Recent” categories due to low number of responses in the “Recent” category. This is in agreement with the lack of recency effect reported in the literature (35) and our dataset. Results indicate no significant difference between the groups in the primacy effects in the delayed recall trial (Table 3), which is in contrast to the impression created by comparing Figures 1C,D (red lines). This discrepancy may result from the large number of “None” responses in the group of patients with AD. Specifically, the serial position curves are calculated as the proportion of each response relative to the total number of participants. Hence, high number of “None” responses diminishes the denominator and may bias the results. Indeed, Table 2 shows that failure to retrieve any word was relatively rare during recall trials 1-3; yet was frequent in delayed recall, particularly in the AD group. Statistical test confirmed that the groups significantly differed in the proportion of “None” responses as compared to all other types of recall. Figure 2A shows the relative probabilities of “First,” “Middle-list,” and “Recent” categories, excluding “None” responses, relatively to a uniform distribution (the leftmost bar in Figure 2). The proportion of “None” response in each group and recall trial is presented in Figure 2B.

Finally, we evaluated the added contribution of the first word recalled in the first recall trial for the differentiation between patients with AD from MCI above the standard measure of the total number of words recalled in the first recall trial. To this end, a nested binary logistic regression with a two-block model was performed: the total number of words recalled in the first recall trial (block 1); the latter plus the identity of the first word recalled in the first recall trial (block 2). The model was statistically significant. The likelihood ratio chi-square test of the total number of words recalled, was significant [$\chi_{\left(1\right)}^{2}$ = 37.569, *p* < 0.0001]. The likelihood ratio chi-square test for the identity of the first word recalled added in the second block was also significant [$\chi_{\left(1\right)}^{2}$ = 2.869, *p* < 0.045, 1-tailed]. The two-block model explained 27.8% of the variance (Nagelkerke *R2*) and correctly classified 70.5% of cases, with a sensitivity of 69.3 and selectivity of 77.1.

### Temporal Contiguity Measures in Delayed Recall

**Figure 3:**
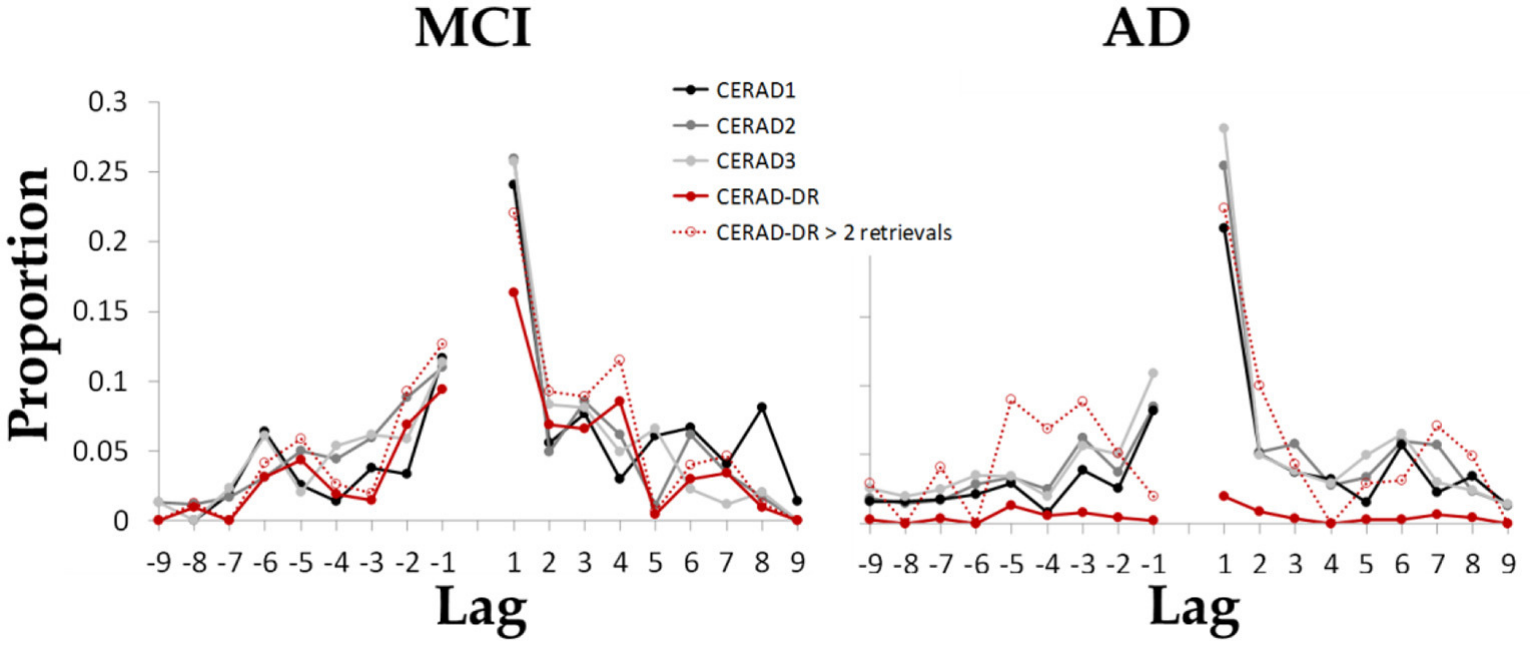
The mean proportion of each possible transition between items as a function of the lag between items in the encoded wordlist. Results indicate that both groups maintained the tendency to retrieve items from nearby serial position of the just-recalled word, as well as the typical asymmetry reflected as greater proportion of forward transitions as compared to backward transitions. Dashed red line present data from a sub group of patients that retrieved at least two items during delayed recall (*N* = 17 patients with AD, 26 patients with MCI).

The continuity curves of the two groups of patients present the known features of lag-CRP curves (Figure 3). In contrast to our hypothesis, the proportion of nearby forward transitions (i.e., +1) and the transitive association index were not significantly lower among patients with AD [*t*_(41)_ = 0.098 *p* = 0.54 and *t*_(29)_ = −1.52 *p* = 0.14, respectively]. As hypothesized, the proportion of nearby backward transitions (i.e.,−1) was significantly lower among patients with AD [*t*_(41)_ = −1.492 *p* = 0.041].

## Discussion

Differentiating between patients with AD and MCI is a challenge that requires converging evidence from multiple sources, tests and measures. Our findings indicate that compared to patients with MCI, patients with AD are: (1) less prone to begin retrieval in the first recall trial with the first word in the wordlist (Table 3); (2) more prone for a total recall failure during delayed recall (Figure 2B); and (3) show reduced backward transitions during delayed recall (Figure 3). Together, these findings indicate that both recall initiation and the dynamics of the retrieval process are affected in AD and that this impairment is evident during both immediate and delayed recall. We suggest that this information has the potential to improve differential diagnosis of MCI and AD.

In the present study, primacy was defined as the very first item in the wordlist since it is unique and qualitatively different from the following items (23, 33) and its recall is influenced by cognitive abilities other than episodic memory *per-se* (36). Our finding of additional predictive value for recalling the first item as the first recall in the first trial, above and beyond the measure of the total number of words recalled in that learning trial, corroborates this notion. Thus, this simple and straightforward measure, which is easily acquired from routine clinical exams, may improve the differential diagnosis between MCI and AD and assist clinicians in their everyday work.

The finding of reduced primacy effect in immediate recall among patients with AD resembles the effect of list length in healthy individuals: the longer the list, the higher the tendency of people to start recall with one of the last serial positions instead of the first ones (26). It is possible that a wordlist that healthy people would consider as relatively short, AD patients would experience as long due to their cognitive impairments. Therefore, in this list they demonstrate an effect similar to the effect healthy adults show in longer lists. Our findings further indicate that the differences between the groups diminish with learning.

Bayley et al. (14) showed similar levels of impairments in primacy effect in the first recall trial for both mild and very mild AD patients, while our MCI group had stronger primacy effect than the AD group. Several factors may contribute to this discrepancy. First, Bayley et al. (14) used the California Verbal Learning Test, in which words are semantically related, and we used the CERAD, in which words are not semantically related. Second, Bayley et al. (14) examined the total number of words recalled, while we focused on the first word recalled.

The current study adds to previous studies (8, 9, 16–18) by characterizing the serial position effect at different stages of learning. We showed that the most pronounced difference between patients with MCI and AD is in the probability of primacy effect at the first recalled word in the first recall trial. In the first recall trial, patients with MCI tend to start recall with the very first item on the list. With learning, some of them retrieve the last items first, although the overall probability to retrieve the first item does not change. Together, these results suggest that with learning, the first item is embedded in memory and the urgency to begin the retrieval with it diminishes. This pattern is not evident among patients with AD, who constantly show reduced primacy effect across all trials. These findings point to long term memory abilities as the source of groups' differences. Neuroscience studies indicate that the hippocampus is critical to long term memory (37) and that it participates in primacy effect (38–42). Therefore, the pattern of memory deficits observed here is in line with the morpho-functional damage seen even in prodromal AD (43, 44).

The finding of increased rate of total delayed recall failure in AD is in line with previous studies that show that delayed recall distinguishes between healthy older adults and dementia patients (45) and between healthy older adults, MCI and dementia (46). Interestingly, though the total number of words retrieved in the delayed recall trial was the most differentiating factor, the serial position effects in that trial did not differentiate the groups. In terms of recall dynamics, compared with MCI, patients with AD had reduced proportion of backward transitions, implying abnormal transition between words during retrieval (Figure 3).

Concerning recency, since patients with dementia or MCI typically demonstrate spared recency effect up until severe dementia (14), it may explain the null recency effect in our study.

Our study has several limitations. First, the CERAD test was administered using a fixed order of 10 words, in a protocol that is best suited to the clinical purpose and the studied population. Shorter length and fixed order of wordlists enhance primacy effect (1). Hence, these two features of the current study hinder generalization. Nevertheless, one must keep in mind that employing longer wordlist and/or changed order when examining patients with dementia would most likely yield a floor effect, rendering the test insensitive. Moreover, the measure that was eventually added to the differentiation between the groups was the first word recalled in the first recall trial. When focusing on the first recall trial, it does not matter whether the following learning trials are administrated in the same or different order. Second, due to our limited sample size, we were not able to compare the very first word to other, non-first, primacy effects. Future studies with larger sample size may clarify this point. Another limitation to our study is the lack of biomarkers for the diagnosis of MCI pathology. During the study period we did not use biomarkers for MCI diagnosis. In future work, as biomarkers will become more available, it will be interesting to see whether the phenomenon described herein is more prevalent in MCI due to AD pathology.

In summary, the first word recalled during the first recall trial contributes to the differential diagnosis between MCI and AD. This information is readily available in wordlist learning and recall tasks and has the potential of improving the specificity of diagnosis at single-patient level and medical care. Future studies are needed in order to establish the usage of the first word recalled during the first recall trial at the single subject level in routine clinical exams. Whether early, non-first, item might show some advantage in comparison with middle-list items should also be explored. Moreover, incorporating the first word recalled with other measures, such as recognition performance and total number recalled may achieve better differentiation between MCI and AD and should be further explored.

## Data Availability Statement

The datasets presented in this article are not readily available because These data is from clinical assessments of Alzheimer's disease (AD) and mild cognitive impairment (MCI) and therefore cannot be published or shred. Requests to access the datasets should be directed to IS-L, iritlichter@yahoo.com.

## Ethics Statement

The studies involving human participants were reviewed and approved by local Institutional Review Boards of Rabin Medical Center and at the Rambam Health Care Campus. Written informed consent for participation was not required for this study in accordance with the national legislation and the institutional requirements since this is a retrospective study.

## Author Contributions

IS-L substantial contributions to the conception and design of the work, analysis and interpretation of data, and writing the work. NO contributed to the analysis and revising the work critically. AA, RB-H, TF, and JAP conducted neuropsychological assessments and evaluation of the patients. AG contributed to the conception of the work and revising the work critically. All authors final approval of the version to be published and agreement to be accountable for all aspects of the work.

## Conflict of Interest

The authors declare that the research was conducted in the absence of any commercial or financial relationships that could be construed as a potential conflict of interest.
